# Identification and Validation of a Novel Antibacterial Compound MZ-01 against Methicillin-Resistant *Staphylococcus aureus*

**DOI:** 10.3390/antibiotics11111550

**Published:** 2022-11-04

**Authors:** Junshu Yang, Christopher Brown, Wayland Noland, Timothy J. Johnson, Yinduo Ji

**Affiliations:** 1 Department of Veterinary and Biomedical Sciences, University of Minnesota, St. Paul, MN 55108, USA; 2 Department of Chemistry, University of Minnesota, Minneapolis, MN 55454, USA

**Keywords:** antimicrobial agents, Gram-positive bacterial pathogen, *Staphylococcus aureus*, MRSA

## Abstract

The discovery of new classes of antibiotics is slow, and it is being greatly outpaced by the development of bacterial resistance. This disparity places us in an increasingly vulnerable position because we are running out of safe and effective therapeutic options to treat antibiotic-resistant infections. This is exemplified by the emergence and persistence of hospital-acquired and community-associated methicillin-resistant *S. aureus* (MRSA), which has markedly narrowed our options for treating life-threatening staph infections. Thus, there is an urgent need to develop novel, potent, preventive, and therapeutic agents. In our current study, we performed a whole-cell screening assay of synthetic libraries for antibacterial activity and identified a novel molecule, MZ-01. MZ-01 exhibited potent bactericidal activity against Gram-positive bacterial pathogens, including MRSA, *Streptococcus pyogenes*, and *Streptococcus pneumoniae,* at low concentrations. MZ-01 killed and lysed both the late exponential phase of an *S. aureus* population and bacteria inside mammalian cells. Furthermore, MZ-01 exhibited low cytotoxicity. These results indicate that MZ-01 is a promising scaffold to guide the development of novel, potent antibacterial agents against multidrug-resistant Gram-positive bacterial pathogens such as MRSA.

## 1. Introduction

Bacterial resistance to antibiotics is a serious threat to public health worldwide. *Staphylococcus aureus* is one of six major pathogens, including *Enterococcus faecium*, *S. aureus*, *Klebsiella pneumoniae*, *Acinetobacter baumannii*, *Pseudomonas aeruginosa*, and *Enterobacter* spp., that are responsible for the majority of antibiotic-resistant infections in the US [1,2,3], and result in tens of millions of infections annually [4]. The persistence of multidrug-resistant *S. aureus*, especially methicillin-resistant *S. aureus* (MRSA), is a serious public health concern [5,6]; in 2017, MRSA caused nearly 20,000 deaths in the US (CDC, 2019). MRSA isolates are responsible for at least 40% of all hospital-acquired MRSA (HA-MRSA) infections [7], whereas the more virulent community-acquired MRSA (CA-MRSA) has emerged and spread worldwide [8,9,10].

In addition to being a concern for human health in healthcare and community settings, significant concerns regarding MRSA in food animals have emerged after the identification of MRSA among Dutch swine farms in 2005 [11]. Livestock-associated MRSA (LA-MRSA) isolates are genetically distinct from human isolates [12]. Most LA-MRSA from swine can be assigned by multilocus sequence typing (MLST) to a single sequence type, ST398 [13]. ST398 MRSA has been detected on many pig farms in different countries, including the US [14,15,16,17]. In the US, 49% of the animals and 45% of the workers examined on farms in Iowa and Illinois carried ST398 MRSA [18]. People working in these barns or in close contact with pigs are at significant risk for ST398 LA-MRSA colonization [12,16,18,19,20,21]. As such, these individuals are at increased risk of developing drug-resistant *S. aureus* infections, and sporadic cases of serious disease have been reported [13,22,23,24]. Furthermore, ST398 MRSA has been found in retail meat products, including ground pork and ground beef, in Europe, Canada, and the US [25,26,27,28]. The rise of MRSA in food animals increasingly threatens food safety and public health because LA-MRSA isolates are now emerging as causes of human disease and death [29,30,31,32].

Several antibiotics, including linezolid and daptomycin, are approved for the treatment of MRSA and are still part of our first and second lines of therapy; vancomycin, linezolid, and clindamycin are used for the treatment of MRSA pneumonia [33]. However, there has been a rise in multidrug resistance against these antibiotics, which further limits our therapeutic options [34,35,36,37,38,39,40]. There are several new antibiotics in the pipeline, but they are mostly limited to existing classes and thus have cross-resistance, which highlights the need for the development of novel antibiotic classes [41,42].

As part of our ongoing efforts to identify new therapies against *S. aureus*, we performed a whole-cell screening assay of synthetic libraries and identified a novel compound: a 3,5-dinitroindole-2-carboxylate derivative, termed MZ-01. In this study, we validated the compound MZ-01 in vitro. MZ-01 exhibited potent bactericidal activity against Gram-positive bacterial pathogens, including *S. aureus* (e.g., MRSA), *Streptococcus pyogenes*, and *Streptococcus pneumoniae* at low concentrations. Yet, even at high concentrations, MZ-01 did not kill Gram-negative bacteria *Pseudomonas aeruginosa* and *Klebsiella pneumoniae.* Importantly, MZ-01 had low cytotoxicity against eukaryotic cell lines. Overall, these results suggest that MZ-01 is a promising compound for the development of selective antibacterial agents for Gram-positive bacterial infections.

## 2. Results

### 2.1. MZ-01 Had Potent Antibacterial Activity against Gram-Positive Bacterial Pathogens, Including S. aureus (e.g., MRSA), S. pyogenes, and S. pneumoniae

In order to identify new antibacterial agents, we screened a small compound library (approximately 5000 compounds) and found that MZ-01 exhibits bactericidal activity at low concentrations against MRSA WCUH29 (MIC: 2.67 μg/mL) and against the clinical human *S. aureus* isolates (MIC50: 4 μg/mL), including USA100 HA-MRSA NRS382, USA200 HA-MRSA NRS383, USA300 CA-MRSA NR384, USA400 CA-MRSA MW2, and methicillin-sensitive *S. aureus* (MSSA) (Table 1). Moreover, MZ-01 showed bactericidal activity against Gram-positive *S. pyogenes* and *S. pneumoniae* (MIC: 5.33 μg/mL), weak antibacterial activity against Gram-positive *M*. *abscessus*, and no activity against Gram-negative *P. aeruginosa* and *K. pneumoniae* at 128 μg/mL (Table 1).

MZ-01 also appeared to have weak activity against *E. coli* at 106.67 μg/mL; however, null mutation of the AcrAB efflux pump increased the susceptibility of *E. coli* to MZ-01 (Table 1), suggesting a specific mechanism of action. These results suggest that MZ-01 has the potential to be developed into a Gram–positive-selective or broad-spectrum antibacterial agent.

### 2.2. MZ-01 Had Potent Bactericidal Activity against MRSA

**Figure 1:**
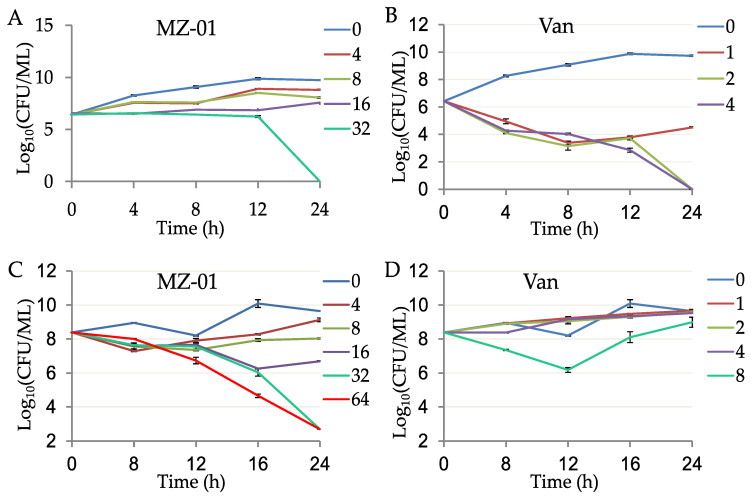
Time-dependent killing of MRSA by MZ-01. MRSA WCUH29 was grown in TSB to early (**A**,**B**) and late (**C**,**D**) exponential phase and were exposed to different doses of antibacterial agents. An aliquot of the culture was taken from each tube at different time points after exposing to the compounds, and a serial dilution was performed. The diluted culture was incubated at 37 °C, and the average values of Log_10_ CFU/mL were determined from triplicate experiments. The unit of MZ-01 and Van (vancomycin) is μg/mL.

To further evaluate the antibacterial activity of MZ-01, we performed time-dependent killing assays by adding different doses (2, 4, 8, 16, 32, or 64 × MIC) of MZ-01 into the early and late exponential phases of an *S. aureus* population, respectively. MZ-01 exhibited potent bactericidal activity against MRSA WCUH29 in a dose-dependent manner (Figure 1A,C); after 24 h exposure to approximate 6.4 log_10_ CFUs of the early exponential phases of *S. aureus*, no viable bacteria were recovered, indicating that MZ-01 at 32 × MIC concentration could kill the bacterial population. In this experiment, Vancomycin (Van) was used as a positive control. Vancomycin killed WCUH29 bacterial cells at 4 to 8 × MIC after 24 h of exposure to the early exponential phase of culture (Figure 1B). Notably, 16 to 32 × MIC of MZ-01 effectively killed and lysed the *S. aureus* population in late exponential phase after 24 h of exposure to WCUH29, whereas more bacteria were viable after exposure to 16 × MIC of vancomycin for 24 h (Figure 1D).

### 2.3. MZ-01 Induces Low Frequency of Resistant Mutations

To determine the frequency of bacterial resistance to MZ-01, we harvested the bacterial cells from the stationary growth of MRSA (WCUH29 strain) and spread them onto TSA plates containing 8 × MIC, 4 × MIC, or 2 × MIC of MZ-01, and incubated for 48 h at 37 °C. We were unable to obtain mutants of *S. aureus* resistant to MZ-01 even when plating 10^10^ CFU onto the media containing 8 × MIC and 4 × MIC of the compound. Only six colonies of *S. aureus* were obtained when plating 10^10^ CFU onto TSA plates with 2 × MIC of MZ-01 after 48 h of incubation at 37 °C. The frequency of resistance to MZ-01 is therefore estimated to be between 10^−9^ and 10^−10^. In addition, only two colonies appeared in 2-fold increased MIC, whereas the rest of the colonies showed similar MIC compared to the control, suggesting that MZ-01 may have multiple targets.

### 2.4. MZ-01 Has Low Cytotoxicity

**Figure 2:**
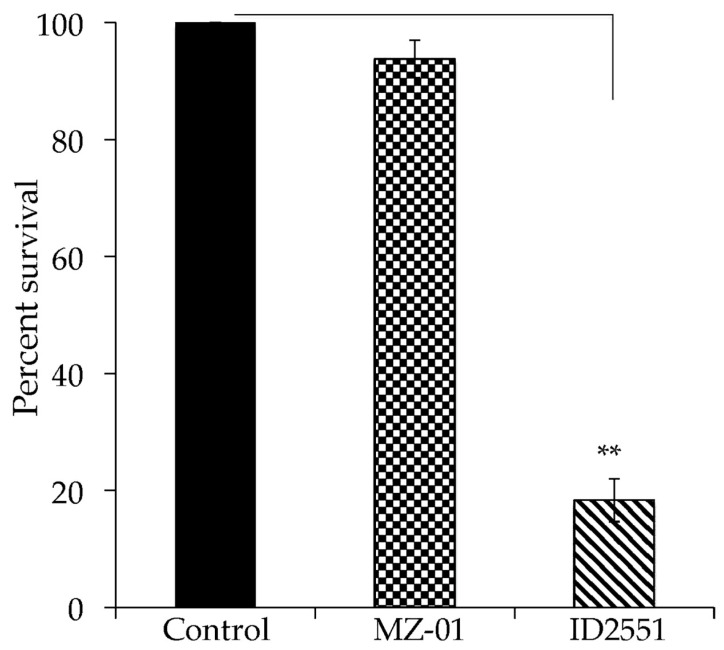
Effect of compounds on cell viability. The monolayer of Vero cells was cultured in RPMI 1640 with 10% FBS and exposed to 400 μM of compounds in DMSO. Control cells were exposed to DMSO vehicle control. Cell viability was measured after 24 h treatment and is expressed as an average of at least three experiments ± standard deviation. The symbol ‘**’ indicates a significant difference between the control and treated cells (*p* < 0.01).

To validate the lead compound, we conducted cytotoxicity assays using two mammalian cell lines, Vero monkey kidney cells, and A549 human lung cancer epithelial cells, in triplicate experiments as described [43]. The cells were incubated in tissue culture flasks at 37 °C in a 5% CO_2_ atmosphere until a confluent monolayer was achieved. The cells were exposed to different doses of compounds for 24 h. Cell viability was determined using the CellTiter 96^®^ Aqueous Non-Radioactive Cell Proliferation Assay; DMSO was used as a negative control. MZ-01 induced less than 5% Vero cell death at 117 μg/mL (400 μM), whereas a positive control compound, ID2551, caused more than 80% cell death (Figure 2).

### 2.5. MZ-01 Kills S. aureus Inside of A549 Epithelial Cells

**Figure 3:**
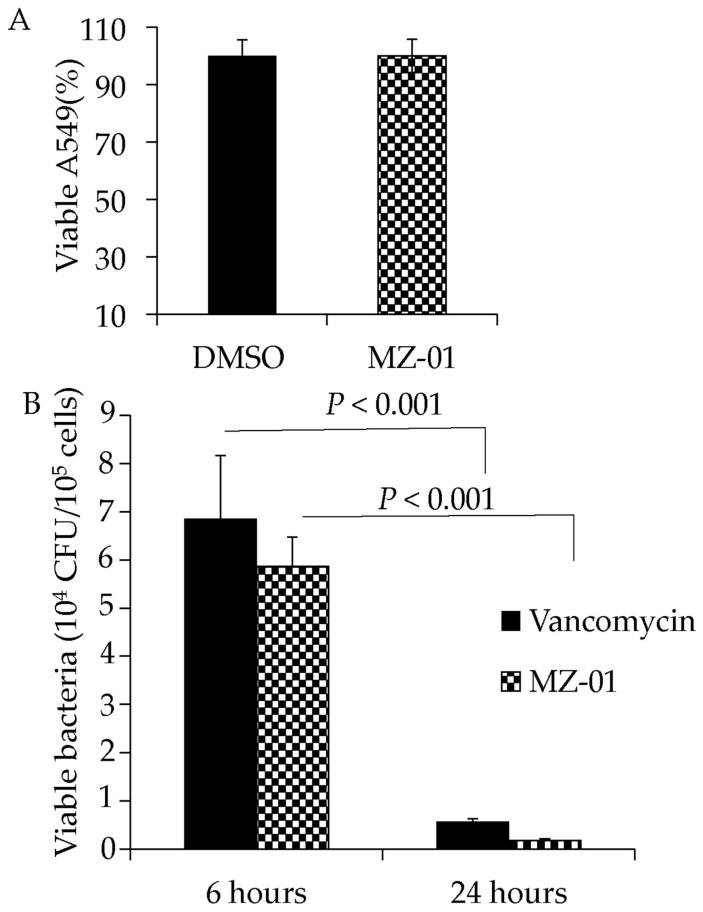
Examination of cytotoxicity and antibacterial activity in human lung epithelial A549 cells. (**A**) Effect of MZ-01 on the viability of A549 cells. The epithelial cells (A549) were incubated in RPMI 1640 medium with 10% FBS. Monolayers of A549 cells (2 × 10^5^ cells well^−1^) were exposed to MZ-01 in DMSO. Control cells were exposed to DMSO vehicle control. Cell viability was measured after 24 h treatment and is expressed as an average of at least three experiments ± standard deviation. (**B**) Recovery of viable MRSA from A549 cells at different time points after treatment with MZ-01 or control vancomycin. Monolayers of A549 cells (2 × 10^5^ cells well^−1^) were infected by approximately 3 × 10^6^ cfu of WCUH29. The extracellular bacteria were removed 1 h after infection. Cell viability was measured at different time points after treatment and is expressed as an average of at least three experiments ± standard deviation.

To assess the activity of MZ-01 in vivo, we examined the efficacy of MZ-01 against MRSA in epithelial cells. A549 cells were infected with MRSA; 1 h after infection, the extracellular bacteria were killed with gentamicin/lysostaphin as described [44]. Infected cells were washed, exposed to 100 μg/mL MZ-01 or vancomycin, and collected in RPMI1640 with 10% FBS 6 or 24 h after treatment. The cells were then lysed, diluted, and plated onto TSA plates to determine viable CFU. No morphological changes were observed in cells after treatment with different antibiotics, including vancomycin. Moreover, 24 h after exposure to the same concentration of MZ-01, A549 cells exhibited no remarkable difference compared to DMSO control (Figure 3A). However, similar to vancomycin, MZ-01 caused significantly decreased viable bacterial cells (MRSA WCUH29) inside of A549 cells 24 h after treatment compared to those treated for 6 h (Figure 3B).

### 2.6. Identification of Key Groups for Activity of MZ-01

To identify key functional groups for MZ-01 activity, we performed pilot SAR studies. Six analogs were selected to identify sites amenable to modification and were examined against MRSA (Table 2). N-methylation at the indole in MZ-04 abolished activity against MRSA compared to MZ-01 and MZ-03. Repositioning of the C5 nitro group to either the C4 or C7 position in MZ-05 and MZ-06, respectively, was not tolerated compared to MZ-01 (Table 2). Removal of the C3 nitro group completely abolished activity in MZ-07, whereas removal of the C5 nitro group in MZ-02 still led to activity that was far less potent than the activity of MZ-01. The shown MIC values are the averages from triplicate experiments.

## 3. Discussion

In this study, we employed the whole-cell screening assays of the compound library collected by Dr. Noland’s laboratory and identified a novel compound, 3,5-dinitroindole (MZ-01) [45,46], with moderate bactericidal activities against Gram-positive bacterial pathogens, including MRSA, low cytotoxicity to eukaryotic cells, and low resistance frequency in MRSA. Altogether, these findings suggest that MZ-01 has the potential to be developed into a potent anti-Gram-positive bacterial pathogen agent. Furthermore, our data showed that MZ-01 possesses weak activity against Gram-negative bacteria *E. coli* and *A. baumannii*, suggesting that it may be possible to expand its spectrum. In addition, the effect of efflux pump (acrAB) deletion on MZ-01′s MIC for *E. coli* is fairly small, which is a desirable characteristic.

MZ-01 possesses a biologically significant indole backbone [47] with two aromatic nitro groups. Although nitroaromatic groups are generally undesirable in drug discovery because of toxicological problems resulting from the metabolic reduction of the nitro group to the corresponding amine [48], some nitroaromatic compounds are used as anti-infection drugs (chloramphenicol, PA-824, metronidazole, and nitrofurantoin), as well as to treat trypanosomatid disease, helminth infections, Parkinson’s disease, angina, and insomnia [49,50,51,52,53]. Taken together, our pilot studies and previous literature strongly support that 3,5-dinitroindole-2-carboxylate derivatives have a remarkable potential to be developed into a new class of antibacterial.

The target of MZ-01 remains to be determined, and we are in the process of identifying the potential mechanism of action for MZ-01 using whole-genome sequencing-resistant mutants and transcriptomics analysis. Null mutation of efflux pump protein AcrAB increased *E. coli* susceptibility to MZ-01, suggesting a specific mechanism of action. We are in the progress of determining its mechanism of action by whole-genomic DNA sequencing analysis of resistant mutants and examining the impact of MZ-01 on global gene transcription profiles using RNA-Seq technologies. It has been reported that the use of nitroaromatic compounds as anti-infective drugs (chloramphenicol, PA-824, metronidazole, nitrofurantoin, etc.) are often prodrugs and their mode of action is involved in bacterial nitroreductases and/or oxidoreductases [54,55,56]. Our results revealed that the frequency of resistance (FOR) is low, less than 10^−9^ in *S. aureus*. This suggests that if activation is required for MZ-01 to inhibit bacterial growth by any enzymes, the enzyme is likely necessary for growth.

Preliminary SAR activity of several pairs of analogs (MZ-01 vs. MZ-02; MZ-03 vs. MZ-04; MZ-01 vs. MZ-07) provides some insights into medicinal chemistry options, which can be analyzed with in silico methods to design new analogs. Meanwhile, additional analogs can be purchased from molecular diversity vendors to augment the internal medicinal chemistry initiatives. These analogs will enable us to perform detailed structure-activity relationship studies in vivo.

Antibacterial drug discovery is extremely difficult because of poor cellular accumulation of compounds due to limited penetration and/or active efflux [57,58]. Although the availability of numerous bacterial genomes and advanced bioinformatics technologies provides powerful tools for identifying a variety of new drug targets, target-directed rational drug design has not been fruitful in antibacterial drug discovery [58]. With results from more than 20 years of study, researchers still have limited success in transforming novel lead compounds into drugs [57,58]. Through our pilot studies, we have demonstrated that MZ-01 is a promising compound that will require further studies to elucidate its mechanism of action and to conduct a detailed SAR analysis and synthesize analogs to further improve its antibacterial activity.

In conclusion, we identified that MZ-01 possesses good antibacterial activity against Gram-positive bacterial pathogens, including MRSA. Our finding suggests that MZ-01 is a promising compound for the development of selective antibacterial agents for Gram-positive bacterial infections.

## 4. Materials and Methods

### 4.1. Bacterial Strains, Plasmids, and Growth Media

*S. aureus* strains used in this study include MRSA isolates USA100 HA-MRSA NRS382, USA200 HA-MRSA NRS383, USA300 CA-MRSA NR384, USA400 CA-MRSA MW2, and 1371 (USA300), and methicillin-sensitive *S. aureus* MSSA isolate MSA553. USA300 (1371) and MSA553 isolates were kindly provided by Drs. Richard Goering and Patrick Schlievert, respectively. The *S. aureus* cells were cultured in Trypticase soy broth (TSB) at 37 °C with shaking. *S. pyogenes* 90-226, *S. pnuemoniae* N1387, and *E. faecalis* V583 strains were kindly provided by Drs. Paul Cleary and Gary Dunny. *M. abscessus* (ATCC 19977) and *A. baumannii* (ATCC 19606) were kindly provided by Dr. Michio Kurosu. *E. coli* (ATCC25922), *K. pneumonia* (ATCC13883), and *P. aeruginosa* (ATCC 27853) were kindly provided by Dr. Christine Salomon. *E. coli* MG1655 wild type and *acrAB* knockout mutant strains were kindly provided by Dr. Vincent Tam.

### 4.2. Antibiotics and Chemical Compounds

Antibiotics, including amoxicillin, chloramphenicol, erythromycin, and vancomycin, were purchased from Sigma-Aldrich. MZ-01, MZ-02, MZ-03, MZ-04, MZ-05, MZ-06, and MZ-07 compounds were from Dr. Noland’s laboratory. These compounds were synthesized as previously described [45,46].

### 4.3. Eukaryotic Cell Culture

Vero monkey kidney epithelial cells (ATCC CCL-81) were cultured in RPMI 1640 medium supplemented with 10% fetal bovine serum (FBS; Invitrogen, CA, USA). Cultures of Vero cells were maintained in a medium containing penicillin (5 µg/mL) and streptomycin (100 µg/mL) (Invitrogen, CA, USA). Assays were performed in RPMI 1640 medium with different doses of tested compounds. A549 cells were cultured in RPMI 1640 as described [43,44].

### 4.4. MIC and MBC Assays

*S. aureus* strains were grown in Trypticase soy broth (TSB) at 37 °C overnight and were diluted to ~10^5^ CFU/mL in MHB for MIC assays with a 96-well microtiter format. Serial dilutions of the compounds were prepared in MHB broth in a final assay volume of 100 μL. Fifty microliters of 10^5^ CFU/mL bacteria were added to the serially diluted antibiotics. The MIC was the concentration at which the antibiotic prevented turbidity in the well after incubation for 18 h at 37 °C, as described [59]. The MBC assay was conducted by dropping 10 μL of overnight culture (from the wells with 4×, 2×, and 1 × MIC in the 96-well plates of MIC assay) onto TSA. The MBC was the concentration at which the antibiotic killed the bacterial cells in the well after incubation for 18 h at 37 °C. The MIC and MBC assays were repeated at least three times, respectively.

### 4.5. Kinetic Time-Killing Assays

Kinetic time-killing assays for antimicrobial agents were conducted based on the CLSI guidelines. The MRSA WCUH29 strain was grown into early and late exponential phase, respectively, in MHB at 37 °C with shaking (225 rpm) and exposed to different concentrations of antibacterial agents. The bacterial solution (50 μL) was collected from the culture at multiple time points and diluted in fresh TSB and plated onto TSA, and incubated overnight at 37 °C for viable CFU. The time-killing assay was repeated at least three times.

### 4.6. Cytotoxicity Assay with Vero and A549 Cells

We conducted cytotoxicity assays using two mammalian cell lines, Vero monkey kidney cells and A549 human lung cancer epithelial cells, in triplicate experiments as described [43]. The cells were incubated in tissue culture flasks at 37 °C in a 5% CO_2_ atmosphere until a confluent monolayer was achieved. Briefly, all cells were grown in 96-well plates to 90% confluence. To test the cytotoxicity, monolayer cells were exposed to different doses of tested compounds and incubated at 37 °C with 5%CO_2_ for 24 h. At the end of the experiment, cell viability was determined using the CellTiter 96^®^ Aqueous Non-Radioactive Cell Proliferation Assay (Promega, MI, USA) as per the manufacturer’s instructions. DMSO was used as a negative control. Each experiment was repeated at least three times, and all of the percentages of cell death related to control were calculated and statistically analyzed by Student’s *t*-test.

To determine the potential antibacterial activity of MZ-01 in vivo, we examined the efficacy of MZ-01 against MRSA in epithelial cells. The human epithelial A549 cells were infected with MRSA. The extracellular bacteria were killed with gentamicin/lysostaphin 1 h after infection, as described [44]. Then, the infected cells were washed and collected at 6 or 24 h after exposure to 100 μg/mL MZ-01 or vancomycin in RPMI1640 with 10% FBS. The cells were lysed, diluted, and plated onto TSA plates for viable CFU.

### 4.7. Data Analysis

Independent samples were statistically analyzed using Student’s *t*-test with an alpha level ≤ 0.05 considered significant.

## Figures and Tables

**Table 1:** Antibacterial profile of MZ-01.

Strain	MIC (μg/mL) *				
	MZ-01	Amoxicillin	Chloramphenicol	Erythromycin	Vancomycin
MRSA WCUH29	2.67	64	6.67	0.5	0.67
USA 300 CA-MRSA JE2	4	ND	ND	ND	ND
MRSA clinical isolates (n = 19)	4 **	ND	ND	ND	ND
MSSA Newman	4	ND	ND	ND	ND
*S. Pyogenes* 90-226	5.33	ND	ND	ND	ND
*S. pneumoniae* N1387	5.33	1.67	2	> 64	0.5
*E. faecalis* V583	>128	64	4	64	>128
*M. abscessus* ATCC19977	128	ND	ND	ND	ND
*E. coli* AcrAB+ ATCC25922	106.7	128	4	85.3	>128
*E. coli* AcrAB−	37.3	32	1.67	8.7	>128
*A. baumannii* ATCC19606	106.7	64	128	21.3	>128
*K. pneumoniae* ATCC13883	>128	>128	6.67	85.3	>128
*P. aeruginosa* ATCC27853	>128	>128	128	128	>128

* Represents average values of MIC obtained from triplicate experiments; ** Represents MIC50.

**Table 2:** Activity of MZ-01 analogs against MRSA WCUH29.

Analog	Structure	MIC (μg/mL) *
MZ-01	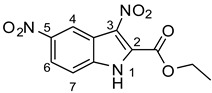	2.67
MZ-02	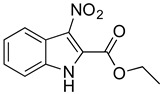	85.33
MZ-03	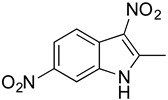	5.33
MZ-04	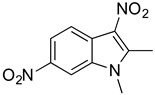	>128
MZ-05	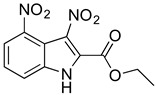	>128
MZ-06	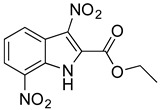	>128
MZ-07	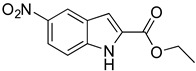	>128

* Average values of MIC from triplicate experiments.

## Data Availability

The data presented in this study are available in the main text, figures, and tables.
